# Dipeptidyl peptidase 1 inhibition as a potential therapeutic approach in neutrophil-mediated inflammatory disease

**DOI:** 10.3389/fimmu.2023.1239151

**Published:** 2023-12-14

**Authors:** James D. Chalmers, Ralph Kettritz, Brice Korkmaz

**Affiliations:** ^1^Department of Molecular and Clinical Medicine, Ninewells Hospital and Medical School, Dundee, United Kingdom; ^2^Department of Nephrology and Medical Intensive Care, Charité-Universitätsmedizin Berlin, Corporate Member of Freie Universität Berlin and Humboldt-Universität zu Berlin, Berlin, Germany; ^3^Experimental and Clinical Research Center, a Cooperation Between the Max Delbrück Center for Molecular Medicine in the Helmholtz Association and Charité Universitätsmedizin, Berlin, Germany; ^4^INSERM UMR-1100, Research Center for Respiratory Diseases, University of Tours, Tours, France

**Keywords:** dipeptidyl peptidase 1, cathepsin C, elastase, neutrophil, inflammation, pathophysiology, treatment strategy, respiratory disease

## Abstract

Neutrophils have a critical role in the innate immune response to infection and the control of inflammation. A key component of this process is the release of neutrophil serine proteases (NSPs), primarily neutrophil elastase, proteinase 3, cathepsin G, and NSP4, which have essential functions in immune modulation and tissue repair following injury. Normally, NSP activity is controlled and modulated by endogenous antiproteases. However, disruption of this homeostatic relationship can cause diseases in which neutrophilic inflammation is central to the pathology, such as chronic obstructive pulmonary disease (COPD), alpha-1 antitrypsin deficiency, bronchiectasis, and cystic fibrosis, as well as many non-pulmonary pathologies. Although the pathobiology of these diseases varies, evidence indicates that excessive NSP activity is common and a principal mediator of tissue damage and clinical decline. NSPs are synthesized as inactive zymogens and activated primarily by the ubiquitous enzyme dipeptidyl peptidase 1, also known as cathepsin C. Preclinical data confirm that inactivation of this protease reduces activation of NSPs. Thus, pharmacological inhibition of dipeptidyl peptidase 1 potentially reduces the contribution of aberrant NSP activity to the severity and/or progression of multiple inflammatory diseases. Initial clinical data support this view. Ongoing research continues to explore the role of NSP activation by dipeptidyl peptidase 1 in different disease states and the potential clinical benefits of dipeptidyl peptidase 1 inhibition.

## 1 Introduction

**Figure 1:**
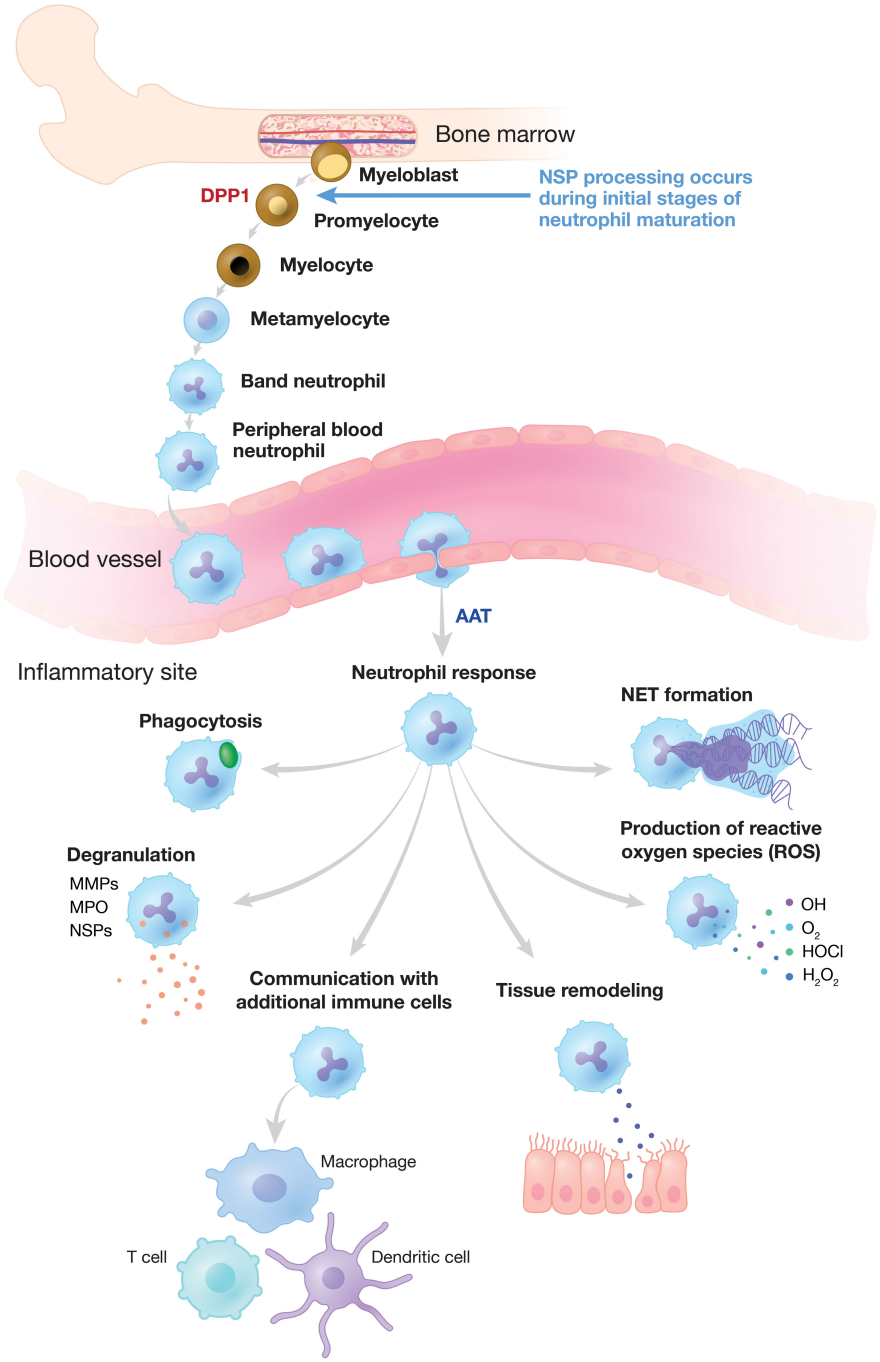
Neutrophilic Response to Inflammation and Infection. NSP proenzymes are activated by DPP1 to their catalytically active forms in promyelocytes during neutrophil differentiation in the bone marrow. Neutrophils migrate via the bloodstream to sites of inflammation and infection where activated NSPs are released and other critical neutrophilic functions are triggered. NSP activity is regulated by endogenous inhibitors (serpins) such as AAT, alpha-1 antitrypsin. DPP, dipeptidyl peptidase; MMP, matrix metalloproteinase; MPO, myeloperoxidase; NE, neutrophil elastase; NET, neutrophil extracellular trap; NSP, neutrophil serine protease.

Serine proteases account for more than one-third of known proteolytic enzymes and are important effectors in many essential physiologic functions, including blood coagulation, digestion, and host immunity (1–3). Serine proteases are synthesized as inactive proenzymes (zymogens) and are locally and transiently activated by regulated proteolytic cleavage in response to specific stimuli (1, 3, 4). Certain immune defense cells, including neutrophils, monocytes, mast cells, and lymphocytes, contain a unique subgroup of serine protease zymogens that are constitutively activated by dipeptidyl peptidase 1 (DPP1; EC 3.4.14.1), also known as cathepsin C (CatC). DPP1 has a principal role in the activation of granule serine proteases (**Figure 1**). These include neutrophil serine proteases (NSPs)—neutrophil elastase (NE), proteinase 3 (PR3), CatG, and NSP4—as well as mast cell chymase and tryptase and lymphocyte granzymes (3–6). Once referred to as leukoproteases (7), the evolutionarily related serine proteases NE and PR3 are CatG homologous NSPs that belong to the chymotrypsin family of proteins (4). Whether activated through limited proteolysis or constitutively during maturation, NSP activity is strictly regulated by endogenous antiproteases (8).

Neutrophils are essential for innate immune defense against invading pathogens and are among the initial mediators of the inflammatory response (9–11). Developed from pluripotent bone marrow stem cells, a tightly regulated reservoir of neutrophils circulates in the bloodstream (12–14). During infection, neutrophils are the first inflammatory cells to leave the vasculature, migrating to sites of inflammation along a chemotactic gradient (**Figure 1**) (14). Neutrophils clear invading pathogens primarily through phagocytosis, after which cytoplasmic granules fuse with the phagosome to mediate pathogen clearance through high concentrations of oxidants, antimicrobial peptides, and proteases (10, 11, 15). Alternatively, neutrophils may attempt to control invading pathogens through the release of neutrophil extracellular traps (NETs). NETs are web-like structures composed of DNA, histones, and granular proteins that physically trap extracellular pathogens (9–11, 16). NSPs are found mainly in neutrophil azurophilic granules and facilitate non-oxidative intracellular and extracellular pathogen destruction (9, 17). The intracellular activity of NE has been reported to be essential for NET formation, and high concentrations of NSPs on NETs are involved in the extracellular degradation of bacterial virulence factors (9, 17, 18).

NSPs participate in immune defenses; they are immunomodulatory, with proteolytic activity in tissue remodeling following injury. However, uncontrolled NSP activity can wreak degradative and inflammatory havoc. Homeostasis between NSPs and their inhibitors—known as the protease-antiprotease paradigm—can be disrupted to the extent that chronic inflammatory lung disease (19) or a myriad of other possible pathologies, including autoimmune disease, blood coagulation defects, cancer, and chronic inflammatory disease, ensue (1, 13). Understanding the mechanisms of neutrophil stimulation and maturation and NSP activation and inhibition is paramount to developing treatments to curtail inflammation and disease.

**Figure 2:**
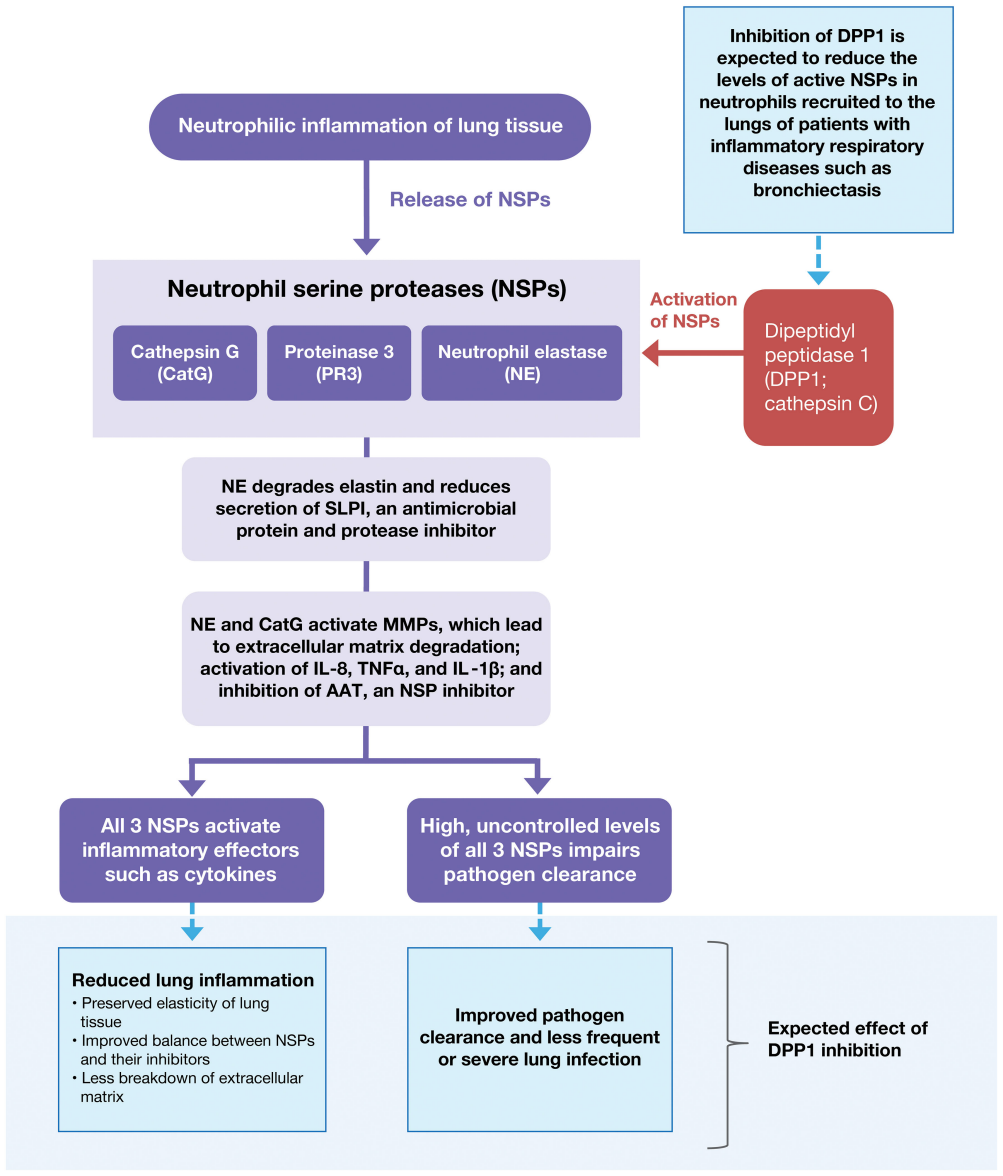
Pathological Spectrum of Aberrant NSP Activity in Lung Tissue. Dysregulated NSP activity contributes to lung damage in multiple acute and chronic inflammatory lung diseases including acute lung injury, acute respiratory distress syndrome, COPD, AATD, bronchiectasis, and CF. Inhibition of DPP1 is under evaluation as a therapy to reverse NSP dysregulation and control the associated inflammation. AAT, alpha-1 antitrypsin; AATD, alpha-1 antitrypsin deficiency; CatG, cathepsin G; COPD, chronic obstructive pulmonary disease; CF, cystic fibrosis; DPP1, dipeptidyl peptidase 1; IL-1β, interleukin-1β; IL-8, interleukin-8; MMP, matrix metallopeptidase; NE, neutrophil elastase; NSP, neutrophil serine protease; PR3, proteinase 3; SLPI, secretory leukocyte protease inhibitor; TNF-α, tumor necrosis factor-α.

The airway is the major interface between the human immune system and the external environment; consequently, the lung is a frequent site of bacterial infection and therefore neutrophil-mediated pathology. Uncontrolled NSP activity contributes to lung damage in multiple acute and chronic inflammatory lung diseases, including acute lung injury, acute respiratory distress syndrome, chronic obstructive pulmonary disease (COPD), alpha-1 antitrypsin deficiency (AATD), bronchiectasis, and cystic fibrosis (CF) (**Figure 2)** (20–23). The adverse effects of uncontrolled NSP activity have been demonstrated in preclinical and clinical studies (8).

The requirement to maintain an adequate immune response to bacterial infection is a key consideration in developing treatments for neutrophil-mediated diseases. Previous attempts to target neutrophilic inflammation in CF, for example, have led to increased rates of infection. In this regard, the loss of NSPs due to the absence of DPP1 is not associated with major immunodeficiency, suggesting that DPP1 is an attractive target for modulation of chronic inflammatory lung disease. For example, individuals with loss-of-function mutations of the DPP1 gene develop prepubertal aggressive periodontitis, Papillon-Lefèvre syndrome (PLS) (24), or Haim-Munk syndrome (25), yet these patients do not exhibit marked immunodeficiency despite the near total loss of active granule-associated NSPs (26). Furthermore, DPP1 knockout mice are protected from NSP-mediated damage (8, 27). Taken together, these findings suggest that DPP1 is an attractive therapeutic target for a number of NSP-mediated pathologies, and DPP1 inhibition is a promising avenue for the treatment of diseases in which neutrophilic inflammation is central to the pathology, such as chronic inflammatory diseases, autoimmune diseases, and cancer. However, achievement of adequate reduction of NSP activity in neutrophils through pharmacological inhibition of DPP1 has been challenging (28, 29). Data suggest that effective attenuation of NSP-mediated damage will likely require the dual inhibition of DPP1 maturation and its aminopeptidase activity (30).

In this review, we provide an overview of DPP1, its functional biochemical properties, the consequences of its inactivation and deficiency, and NSP pathophysiology in a variety of disorders. We conclude with an assessment of the potential of DPP1 as a therapeutic target and current progress in the development and clinical evaluation of DPP1 inhibitors.

## 2 DPP1 biology and pathophysiology

### 2.1 Biosynthesis, processing, and maturation

**Figure 3:**
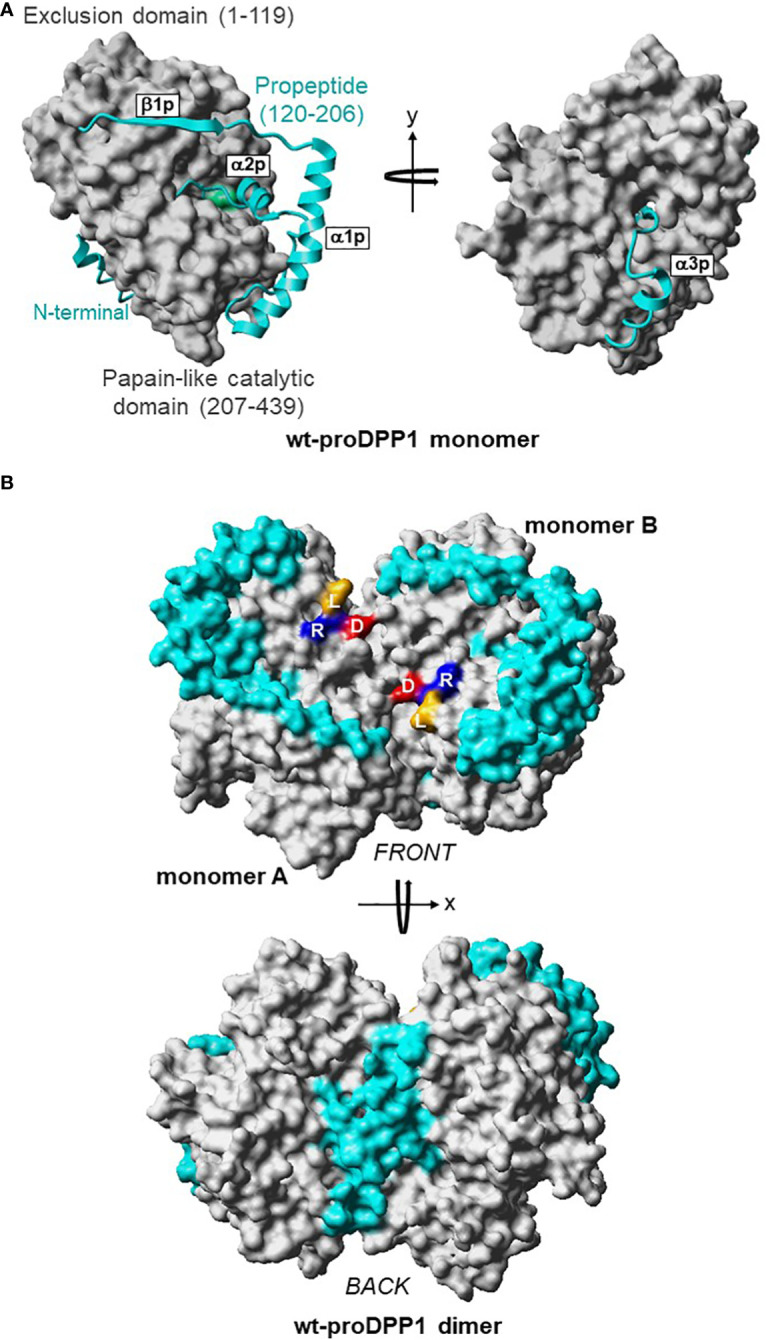
3D Structures of Dimeric Pro-DPP1 and Its Functional Tetrameric Form. Model structure **(A)** DPP1 monomer. The exclusion domain and papain-like catalytic domain are shown in surface representation and colored in grey. The propeptide (residues Thr120–His206) in the ribbon plot is shown in cyan. The catalytic cysteine 234 is shown in green. **(B)** ProDPP1 dimer. Monomers A and B are shown in surface representation with the same color coding as in **(A)**. Leucine, arginine, and aspartic acid are labeled using their one-letter amino acid codes and shown in dark yellow, blue, and red, respectively. Cat, cathepsin; D, aspartic acid; DPP, dipeptidyl peptidase; L, leucine; R, arginine; wt, wild type. Figure previously published in Lamort AS, et al. Int J Mol Sci 2019; 20:4747; reproduced with permission.

DPP1 is expressed ubiquitously in mammals; the highest expression levels are in the lung, spleen, kidney, liver, and myeloid cell lineages, particularly neutrophils, mast cells, monocytes, macrophages, and their precursors (31). DPP1 is a unique member of the papain-like cysteine protease superfamily in terms of its biosynthesis, processing, and quaternary structure (32). Initially synthesized as a single-chain monomeric proenzyme (32–34), DPP1 spontaneously folds into the proDPP1 homodimer containing an exclusion domain, a propeptide, and both heavy and light chains that form a papain-like catalytic domain (**Figure 3**) (32, 33). DPP1 activation is achieved primarily via processing by CatL-like cysteine cathepsins in a two-step process (27, 30, 32, 33).

Crystal structures of DPP1 in complex with small molecule inhibitors have provided insight into the substrate-binding pockets (35–37). The heavy chain of each monomer harbors a catalytic cysteine residue in the solvent-exposed active site (33–35, 38). The active site of each monomer contains a hydrophobic interior pocket and a large surface-exposed pocket, an architecture that accommodates the wide substrate specificity of DPP1 (30, 34, 39–42). Furthermore, the exclusion domain, which is unique to DPP1, is responsible for the exopeptidase activity of this enzyme because it blocks regions of the enzyme active site and makes it accessible only to the N‐terminus of its protein substrates (34). Upon activation, mature serine proteases and DPP1 are stored together in cytoplasmic granules. ProDPP1 is constitutively secreted extracellularly from the Golgi apparatus (30); however, activation of neutrophils, mast cells, and lymphocytes results in the release of active DPP1 from granules into the extracellular milieu (30, 43, 44).

### 2.2 Extracellular DPP1

Upon neutrophil activation, mature DPP1 and other granule-associated proteases are secreted into the extracellular milieu. Mature DPP1 has been found in sputum from patients with CF and asthma (30), in tracheal aspirates from mechanically ventilated patients with pneumonia (45), and in bronchoalveolar lavage fluid from patients with non-small cell lung cancer, including patients with low neutrophil numbers (30). In contrast, proDPP1—but not mature DPP1—is constitutively secreted by bronchial epithelial cells from healthy individuals, positioning mature DPP1 as a biomarker of neutrophilic lung inflammation (30). However, the functional role of extracellular DPP1 secreted from neutrophils at inflammatory sites remains uncertain.

DPP1 is abundantly expressed in mast cells. *In vitro* data suggest that DPP1 is packaged into serine protease–rich mature granules (or a functionally similar compartment) and secreted when mast cells are activated (44). The high expression level of DPP1 and serine proteases in mast cells, neutrophils, and cytotoxic T lymphocytes suggests that curtailing the downstream effects of DPP1 activation may alter many cellular functions, including mast cell function, and impede diseases driven by mast cells.

### 2.3 Genetic inactivation

Genetic inactivation or loss of DPP1 activity can have widespread and variable effects. PLS is a rare autosomal recessive disease in which DPP1 insufficiency, which is caused by a mutation in the DPP1 gene (*CTSC*), results in diffuse palmoplantar hyperkeratosis, severe prepubertal periodontitis, and premature loss of both deciduous and permanent teeth (26, 46). Neutrophil-mediated bacterial killing remains intact in patients with PLS, although neutrophil activity has several functional defects, including impaired chemotaxis, abnormal release of proinflammatory cytokines, and a lack of NET formation (26, 46, 47).

Characterization of NSPs from patients with PLS revealed a DPP1-independent processing and maturating pathway for NSPs that is catalyzed by at least one DPP1-like protease (48) termed NSP-activating alternative protease (NSP-AAP). The proteolytic elimination pathway of proNSPs is catalyzed by an unknown granule-associated protease, a pathway initially identified by the differentiation of isolated human neutrophilic precursors in the presence of the cell-permeable DPP1 inhibitor (28, 48). In this pathway, most proNSPs are eliminated during neutrophil differentiation, while only 5% or less of proNSPs mature (48).

In the human promyelocytic HL-60 precursor cell line, CatS/CatL inhibition nearly abolishes proDPP1 maturation, although it does not result in significant NSP inactivation. It is hypothesized that even in the presence of CatS/CatL inhibition and blockade of proDPP1 maturation, NSP-AAP can activate approximately 80% of NSPs. However, treatment of HL-60 cells with a nitrile inhibitor that reduces both DPP1 and NSP-AAP activities by approximately 95% results in near-complete NSP inactivation (30). Taken together, these data support the action of a yet unknown protease in the human proteome that is involved in proNSP maturation.

Granzymes are serine proteases with various substrate specificities that are expressed almost exclusively in cytotoxic T cells and natural killer cells. Granzyme A, a tryptic protease (49), and granzyme B, an aspartic protease (50), are the most commonly expressed granzymes during T- and natural killer cell activation (51) and are maturated by DPP1 *in vitro*. Lymphokine-activated killer cells from patients with PLS and DPP1 deficiency retain 50-60% of normal granzyme activity and display normal cytotoxic activity against K562 cancer cells (26). These results highlight that DPP1 is not the sole proteinase involved in pro-granzyme maturation in humans. Furthermore, the presence of DPP1-like proteinase(s) provides a molecular rationale for the absence of a generalized T-cell immunodeficiency phenotype in patients with PLS.

## 3 Pathophysiology of NSPs

Once active, NSPs play an integral role in modulating inflammation and tissue remodeling. Uncontrolled NSP activity can cause significant tissue damage; therefore, endogenous inhibition of NSPs is critical to maintain health and limit disease progression (**Table 1**) (13, 80, 81).

**Table 1:** Pathobiology of neutrophil serine proteases in inflammatory diseases.

Disease	Pathophysiology	Neutrophil Serine Protease Involvement	Strength of Evidence*
Chronic obstructive pulmonary disease	Neutrophilic airway inflammation causes progressive destruction of the lung parenchyma (52, 53).	Activated neutrophil serine proteases disrupt the normal protease-antiprotease balance; excess extracellular neutrophil serine proteases can cause pulmonary damage directly (53, 54).	***
Alpha-1 antitrypsin deficiency	Low alpha-1 antitrypsin causes neutrophilic airway inflammation and airflow obstruction, bronchiectasis, impaired bacterial clearance, and other airway abnormalities (55, 56).	Alpha-1 antitrypsin is a principal endogenous neutrophil serine protease inhibitor; low levels can trigger disruption of the normal protease-antiprotease balance (53, 57). Neutrophils and neutrophil serine proteases in lung fluids may be elevated (58).	**
Bronchiectasis	Irreversible bronchial dilatation, pulmonary inflammation, and mucus obstruction lead to recurrent infections and progressive lung damage (59, 60).	Airway inflammation is mediated primarily by excessive neutrophil serine proteases release (60).	***
Cystic fibrosis	Mucus dehydration and airway pH reduction facilitate persistent pulmonary bacterial infections and chronic inflammation, leading to progressive lung damage (61).	Excess neutrophil elastase activity causes lung necrosis and apoptosis by degrading essential proteins and activates release of neutrophil extracellular traps that further increase airway viscosity and contribute to persistent inflammation (59, 61–64).	***
Chronic rhinosinusitis without nasal polyps	Occurs as a common comorbidity of chronic lower respiratory conditions, such as chronic obstructive pulmonary disease or bronchiectasis (65, 66).	Neutrophilic infiltration contributes to rhinosinusitis symptoms; neutrophil elastase activity may trigger inflammation with goblet cell metaplasia (66, 67).	*
Antineutrophil cytoplasmic autoantibodies-associated vasculitis	Autoimmune condition with granulomatosis and/or microscopic polyangiitis; it often involves lungs and kidneys, potentially resulting in severe pulmonary hemorrhage and renal failure (68).	Antineutrophil cytoplasmic autoantibodies binding to cellular targets, including proteinase 3, activates neutrophils and monocytes; antineutrophil cytoplasmic autoantibodies-activated neutrophils release neutrophil extracellular traps with neutrophil serine proteases, and neutrophil serine proteases are used by myeloid effector cells to induce necrotizing vasculitis (68, 69).	**
Hidradenitis suppurativa	Inflammatory skin disease characterized by recurrent inflamed nodules, abscesses, sinus tracts (tunnels), and scarring involving the intertriginous regions (70, 71).	Neutrophils recruited to hidradenitis suppurativa lesions contribute to nodule and abscess development; elevated neutrophil elastase and neutrophil extracellular traps occur in patients with hidradenitis suppurativa (70, 71).	*
Cancer	Some cancers are associated with inflammation and tumor-derived immune cells, particularly tumor-associated neutrophils; inflammation may increase the risk of cancer recurrence (72–77).	Immunosuppressive protumor neutrophils foster tumor invasion, metastasis, and angiogenesis by releasing neutrophil elastase and other proteins. Neutrophil serine proteases have been associated with poor clinical outcomes in several types of cancer (72, 73, 78, 79).	**

*Strength of evidence (*weak, **moderate, ***strong) supporting neutrophil serine protease dysfunction as a key etiologic factor.

Proteolytic action of NSPs is controlled endogenously by the serpin (serine proteinase inhibitor) superfamily of proteins (e.g., alpha1-antitrypsin (AAT), alpha1‐antichymotrypsin, monocyte NE inhibitor) and the chelonianin family of canonical inhibitors (elafin, secretory leukocyte protease inhibitor) (8, 82, 83). Serpins include more than 1000 proteins expressed in animals, plants, and viruses (84–86). Serpin-mediated NSP inhibition is unique in that after the initial formation of a non-covalent and reversible serpin-protease complex, two pathways can occur (87). In the inhibitory pathway, the protease cleaves a region of the serpin, generating an irreversible covalent protease-serpin complex; in the substrate pathway, the serpin is irreversibly inactivated after cleavage by the protease, and the protease is regenerated (86). Canonical inhibitors use a standard mechanism to inhibit NSPs, in which a reversible protease-inhibitor complex forms but can slowly dissociate after cleavage of the protein inhibitor (88, 89). Elafin and secretory leukocyte protease inhibitor are synthesized at local sites of injury or in the liver following an inflammatory signal (80) and are two of the main canonical inhibitors that have been reported in human lung secretions (90, 91). Both canonical inhibitors and serpins are important regulators in balancing not just NSP activity, but other serine proteases as well, in both tissues and circulation (92).

### 3.1 Inflammatory respiratory diseases

NSPs are released in the airways of patients with multiple types of chronic pulmonary diseases such as COPD, AATD, bronchiectasis, and CF, and have been implicated as key mediators of chronic inflammation and disease progression (8, 59). Although the etiologies of these conditions vary, excess extracellular NSP activity is a common characteristic. The protease–antiprotease theory for the pathogenesis of COPD has its roots in experimental models of emphysema from the 1960s and the observation that individuals with genetic deficiency of AAT are particularly susceptible to severe emphysema and COPD (3). This theory suggests that the pathogenesis of chronic inflammatory diseases involving NSPs is the result of an imbalance between enzymes that degrade the extracellular matrix within the lung and proteins that oppose this proteolytic activity.

#### 3.1.1 Chronic obstructive pulmonary disease

COPD is characterized by persistent neutrophilic inflammation of the airway lumen and destruction of the lung parenchyma, leading to progressive deterioration of lung function (52, 53). Elevated neutrophil counts have been shown to correlate with progression of COPD in smokers (93) and with acute exacerbations of COPD (94).

Multiple lines of evidence point to NSPs as primary mediators of COPD-associated pulmonary tissue damage and clinical decline (95, 96). In patients with COPD, activation and degranulation of neutrophils result in the release of activated NSPs, primarily NE, PR3, and CatG (54), disrupting the normal balance between proteases and antiproteases (53). Excess extracellular levels of NSPs in COPD have been shown to cause pulmonary tissue damage directly (54). Normally, antiproteases such as AAT limit this damage, but when excessive amounts of NSPs are released in patients with COPD, the controlling effect of antiproteases is overwhelmed (97). In smokers, this situation is exacerbated by the oxidizing environment associated with cigarette smoke, which inactivates AAT (98). Hypoxia also adversely affects neutrophil function in other ways, resulting in reduced destruction of bacterial pathogens and apoptosis (52, 99).

NE has generally been regarded as the principal NSP contributing to the pathophysiology of COPD, but more recent data indicate that PR3 and CatG also have significant roles (97, 100). Studies suggest that free PR3 has a greater radius of activity than NE upon release from granules (101). Some antiproteases produced in the lungs inhibit NE but not PR3, such that PR3 but not NE activity can sometimes be detected in lung secretions (97, 102, 103). Although NSPs can degrade a broad array of structural and other proteins, the specific mechanisms with greatest impact on COPD progression remain unresolved (100).

#### 3.1.2 Alpha-1 antitrypsin deficiency

AAT is the archetypal member of the serpin superfamily (53). AAT, the most abundant antiprotease found in human plasma, is mainly produced in the liver but is also secreted from bronchial epithelial cells (104). AAT has inhibitory activity against all NSPs but is particularly active against NE (57). A primary biological role of AAT is maintenance of the normal protease-antiprotease balance (53).

AATD is an underrecognized genetic condition characterized by low circulating AAT levels that may lead to lung and liver disease; approximately 1 in 2000-5000 individuals are affected (53, 55, 56, 105, 106). AAT deficiency was first described in 1963 when absence of the alpha1 protein in serum was associated with early-onset emphysema in several patients (106). Histological and radiological analyses of affected patients showed severe emphysema and frequently bronchiectasis.

AATD is associated with basal emphysema and features of typical COPD. Elevated neutrophil counts and NSP levels in lung epithelial lining fluid have been reported (58). The airways show evidence of neutrophilic airway inflammation leading to airflow obstruction, bronchiectasis, impaired mucociliary clearance, and impaired bacterial clearance. Patients may be chronically infected with bacteria, contributing to an increased risk of exacerbation. AATD also is associated with panniculitis as well as vasculitis and a 10-fold increase in ulcerative colitis. Finally, emphysema itself is associated with an increased prevalence of lung cancer. All these features have a clear NSP implication and hence are modifiable.

Case reports of adults with AATD have indicated a possible relationship with cutaneous or systemic vasculitis and neutrophilic panniculitis (107, 108). The estimated prevalence of neutrophilic panniculitis is 0.1-0.9% in patients with AATD; these are most commonly white individuals with a Pi*ZZ genotype. Panniculitis presents with painful recurrent subcutaneous nodules that may be ulcerating and is characterized by the presence of dense neutrophils in the deep dermis and connective tissue septae (108, 109).

#### 3.1.3 Bronchiectasis

Bronchiectasis is a heterogeneous condition defined by irreversible bronchial dilatation, pulmonary inflammation, mucus obstruction, and progressive lung damage (59, 60). Recurrent bacterial infections are common and have been proposed as causal elements of a pathogenic vortex in which infections lead to chronic inflammation that in turn increases vulnerability to further infection. Predisposing conditions associated with bronchiectasis include severe infections; immunodeficiencies; hypersensitivity conditions, such as allergic bronchopulmonary aspergillosis; autoimmune conditions; and congenital diseases, such as primary ciliary dyskinesia.

Inflammation in bronchiectasis is mediated primarily by activated neutrophils that release NSPs (60). Although NE release and subsequent formation of NETs are key components of the innate inflammatory response to pathogens (60, 110, 111), the excessive release of NE that occurs in bronchiectasis contributes to disease progression. In a longitudinal study of patients with bronchiectasis, NE activity in sputum was significantly associated with airway bacterial load, risk of bronchiectasis exacerbations, pulmonary functional decline, and risk of hospitalization (112). Sputum NE activity was shown to be associated with increased risk and frequency of exacerbations, infections, hospitalizations and all-cause mortality (22, 23). Levels of PR3 were found to be raised in patients with bronchiectasis during exacerbations compared with stable disease, correlating with levels of NE (113). CatG activity was also found to cause dysfunction of ciliated cells and destruction of airway epithelium in patients with bronchiectasis, and activity correlated with disease severity (114). These observations provided the rationale for clinical trials of agents designed to reduce NSP activity in patients with bronchiectasis (115, 116). The relationships of bronchiectasis and NE release to infection were supported by the observation that antibiotic treatment significantly reduces sputum NE. Protease-antiprotease balance is extremely disrupted in severe bronchiectasis, as shown in proteomic studies of sputum from patients with bronchiectasis that demonstrated that both elevated NSPs and reduced secretory leukocyte protease inhibitor levels were associated with disease severity (117, 118). However, other studies have shown that antibiotic therapy alone is insufficient to modify disease progression because other factors can maintain inflammatory processes and tissue damage (60).

#### 3.1.4 Cystic fibrosis

CF is caused by a genetic deficiency of the CF transmembrane conductance regulator protein, which is produced primarily in epithelial cells of airway, mucus-producing, secretory tissues (61, 119). CF transmembrane conductance regulator dysfunction results in airway mucus dehydration and reduction in the pH of airway surface fluids; these changes compromise antibacterial defenses and facilitate persistent pulmonary bacterial infections. CF transmembrane conductance regulator dysfunction also impairs immune responses to infection by neutrophils, macrophages, and T lymphocytes (61, 120–122). Together, these processes lead to chronic systemic and airway inflammation, pulmonary tissue damage, and respiratory insufficiency.

In patients with CF, NE participates in pathogenic processes that cause disease progression. NE contributes to production of hyperconcentrated airway mucus and airway obstruction (59, 123, 124). Several processes are involved: NE increases expression of the mucin 5AC gene by both transcriptional and posttranscriptional mechanisms and activates mucin secretion from the bronchial epithelium. NE also decreases ciliary functioning and reduces surface hydration by disrupting regulatory ion channels.

As in other chronic respiratory diseases, excessive NE proteolytic activity causes tissue damage and disease progression by degrading proteins essential for maintaining alveolar structure (59, 62), increasing apoptosis and necrosis in the lungs, and activating senescence in airway epithelial cells (63, 64). NE also activates release of NETs that further increase sputum viscosity and contribute to persistent inflammation (61, 122). As in non-CF bronchiectasis, NE activity in sputum has been shown to be associated with disease severity and to predict lung function decline (125).

#### 3.1.5 Chronic rhinosinusitis without nasal polyps

Chronic rhinosinusitis (CRS), defined by chronic inflammation of the paranasal sinuses, is a common comorbidity in individuals with chronic lower respiratory conditions, such as COPD, bronchiectasis, CF, and asthma (65, 66). Co-diagnosis of bronchiectasis with CRS or other chronic respiratory conditions is associated with more severe disease (65, 126–129). CRS is reportedly present in 51% of patients with COPD and 45-77% of patients with bronchiectasis (127, 130).

CRS is classified phenotypically by the endoscopic presence or absence of nasal polyps, referred to as CRSwNP and CRSsNP, respectively (131); approximately 80% of patients with CRS have the CRSsNP phenotype. CRSsNP histological findings include basement membrane thickening, fibrosis, and goblet cell hyperplasia (66). The risk of developing CRSsNP is increased by respiratory viral or bacterial infections, chronic lung diseases, bronchitis, rhinitis, gastroesophageal reflux disease, immunodeficiencies, and some other conditions.

The pathogenesis and underlying mechanisms of CRSsNP are complex. Overall, multiple endotypes of CRSsNP together describe a mixed inflammatory condition with helper T type 1, 2, and 17 components, although CRSsNP is proportionally more neutrophilic and less eosinophilic than CRSwNP (66). Evidence suggests that neutrophilic infiltration contributes to disease symptoms. *In vitro* data and mouse studies suggest that NE proteolytic activity triggers an inflammatory process that induces goblet cell metaplasia, which results in overexpression of mucins that contribute to the symptomatology of CRS (66, 67).

### 3.2 Antineutrophil cytoplasmic autoantibodies associated vasculitis

Antineutrophil cytoplasmic autoantibodies (ANCA)-associated vasculitides (AAV) are a group of autoimmune systemic small vessel diseases (68). Patients with AAV harbor ANCA to PR3 (132, 133) or myeloperoxidase (MPO) (134), which are both autoantigens exclusively expressed by neutrophils and monocytes. The clinical AAV entities are granulomatosis with polyangiitis, microscopic polyangiitis, and eosinophilic granulomatosis with polyangiitis. The lungs and kidneys are frequently involved, resulting in life-threatening pulmonary hemorrhage and rapidly progressive renal failure often requiring renal replacement therapy. Untreated, systemic AAV is invariably lethal. Standard therapies consist of steroids, cytotoxic drugs, and depleting anti-CD20 antibodies. With these treatments, approximately 75% of patients with active AAV achieve remission at 12 weeks, and 30-50% experience vasculitis flares. Improved treatment efficacy, reduced treatment-related morbidity and mortality, and disease-specific strategies are needed. Recently, C5a receptor blockade was found to be protective in a murine AAV disease model (135) and steroid sparing in patients with AAV (136).

ANCA binding to their target cell surface antigens activate neutrophils and monocytes that subsequently contribute to vascular inflammation and injury. NSPs are used by myeloid effector cells to induce necrotizing vasculitis. Some NSP-dependent mechanisms pertain to both PR3- and MPO-ANCA, whereas others specifically relate to the former. PR3 is a unique NSP family member because it acts as both an ANCA autoantigen and an active serine protease (97).

The presentation of the PR3 autoantigen on the cell membrane (mPR3) of neutrophils and monocytes is pivotal for PR3-AAV. PR3-ANCA bind and cross-link mPR3 (137) on neutrophils, thereby initiating, together with Fcγ receptor engagements (138, 139), intracellular mitogen-activated protein kinase (140), Syk tyrosine kinase (141), and phosphoinositide 3-kinase/Akt (142, 143), signaling and subsequently cell activation. The mPR3 pattern on human neutrophils is bimodal (144), caused by a subset-restricted expression of neutrophil-specific CD177 (145). CD177 binds PR3 with high affinity as shown by ectopic expression studies (146) and surface plasmon resonance (147). Epigenetic CD177 silencing in one neutrophil subset and random monoallelic expression in another neutrophil subset yields two distinct CD177^neg^/mPR3^low^ and CD177^pos^/mPR3^high^ cell populations (148). The latter ranges from 0% to 100% and is stable in a given individual. The higher the CD177^pos^/mPR3^high^ neutrophil percentage, the stronger the PR3-ANCA–induced neutrophil activation *in vitro* (149) and the worse the clinical outcome (150, 151). Thus, PR3 on the membrane of viable neutrophils is highly relevant to PR3-ANCA vasculitis.

ANCA-activated neutrophils either release PR3, human NE, and CatG into the surroundings as soluble molecules or tethered to NETs. Degranulated, proteolytically active NSPs are acquired by endothelial cells and cleave endothelial proteins (152), increasing endothelial permeability and injury. In addition, NETs are detected in patients with AAV. ANCA-activated neutrophils generate NETs that are decorated with NSPs together with other granule proteins, DNA, and histones (69). Human NE participates in NET formation by promoting chromatin decondensation (17). ANCA induce NETs via receptor-interacting protein kinase 1/3–dependent necroptosis, and these NETs provide a scaffold for the activation of the alternative complement pathway that in turn causes endothelial cell damage *in vitro*. The *in vivo* relevance of necroptosis, NETs, and the alternative complement pathway, specifically the C5a-C5a receptor interaction, for disease induction was established in murine MPO-AAV models (135, 153–155).

Two large genome-wide association studies found significant associations between PR3-AAV, but not MPO-AAV, and single nucleotide polymorphisms (SNPs) in the PR3 and AAT genes (156, 157). Some of the PR3 SNPs are associated with higher PR3 plasma levels in healthy individuals (158). Another study found that NSP transcription that is normally silenced during neutrophil maturation is reactivated in blood neutrophils from patients with active AAV (159, 160). Comparison of AAT and PR3 in individuals with or without AAV showed that in patients with PR3-AAV, but not those with MPO-AAV, elevated PR3 pools were quantitatively associated with ANCA titer, inflammatory response, and disease severity (161). These findings suggest that oxidation-resistant AAT or other strategies for reducing PR3 activity may have value as adjunctive therapy for PR3-AAV.

NSP contributions to AAV suggest that NSP downregulation provides protection from AAV. Several strategies to reduce NSPs are conceivable, including pharmacological DPP1 inhibition. A DPP1-targeting approach is supported by the observation that chimeric mice that received DPP1-deficient bone marrow were protected from MPO-ANCA vasculitis (155). Human data from patients with PLS showed abrogated NET (162) formation and strongly reduced mPR3 on activated neutrophils and monocytes (48, 163). Pharmacological DPP1 inhibition in neutrophils differentiated from human hematopoietic stem cells recapitulated these findings (48). Consequently, decreased mPR3 levels resulted in reduced PR3-ANCA–induced neutrophil activation. Moreover, both genetic DPP1 deficiency and pharmacological inhibition reduced neutrophil-induced glomerular microvascular endothelial cell damage (163). Conceivably, DPP1 inhibition is more effective in PR3-AAV because of the importance of PR3 as an autoantigen and mPR3 for the activation of myeloid effector cells by PR3-ANCA. Since no appropriate PR3-AAV model exists, clinical studies with adjunctive DPP1 inhibitor administration in patients with PR3-AAV may provide the best approach to test this hypothesis.

### 3.3 Hidradenitis suppurativa

Neutrophils are also the predominant leukocyte infiltrate in HS lesions. HS is a chronic inflammatory skin disease characterized by recurrent inflamed nodules, abscesses, sinus tracts (tunnels), and scarring involving the intertriginous regions (i.e., the axillae, inguinal, and anogenital area). Neutrophils recruited to HS lesions may play an essential role in the development of the inflammatory nodules and abscesses (70). Elevated levels of both NE and NETs are observed in tissue samples of patients with HS, and NETs from HS lesions are associated with worsening disease (71).

### 3.4 Cancer

The World Health Organization ranks cancer as the second most common cause of death globally. More than 2 million new cases of lung cancer alone were reported in 2020 (164, 165). Lung cancer subtypes and associated metastases have been studied with respect to mutation patterns and molecular disease mechanisms (72, 166, 167). When adhesion molecules are lost, cancer cells can disseminate outside the primary tumor, remaining dormant and undetected until they engender metastases or secondary tumors, sometimes years later. The lungs are a common site of metastases for tumors originating from many other tissues (168). Development and progression of some cancers are associated with inflammation and tumor-derived immune cells, particularly tumor-associated neutrophils (TANs). TANs can have both tumorigenic and antitumorigenic functions, referred to as protumor and antitumor TANs, respectively (72–74).

Antitumor neutrophils interfere with tumor cell proliferation directly as well as by recruiting additional immune cells. Protumor neutrophils, on the other hand, are immunosuppressive and foster tumor invasion, metastasis, and angiogenesis by releasing NE and other proteins, e.g. matrix metalloproteinase-9 (MMP-9), S100 calcium-binding protein A8 (S100A8), S100 calcium-binding protein A9 (S100A9), Bv8, and high mobility group box 1 (HMGB1) (73, 78, 79, 169, 170). Smoking also increases the risk of tumor progression and death in patients with breast cancer (171).

Studies suggest that inflammation may increase the risk of cancer recurrence (75–77). Chemokines recruit neutrophils to tumor sites, creating a microenvironment that favors cancer progression and adverse patient outcomes (172, 173). Many of the details regarding the specific chemokine pathways that trigger neutrophil recruitment to tumor sites remain uncertain (174); however, neutrophils have been identified as key in awakening quiescent cancer cells and promoting cancer-associated thrombosis, which can cause significant morbidity in patients with cancer (73, 175). NETs released by activated neutrophils are covered with proteins, including NSPs (18) and tissue factor (176). The procoagulant and prothrombin effects of NETs suggest that targeting NETs may comprise a viable approach for prevention of cancer-related thrombosis. Preclinical models suggest that cancer can enhance NET formation by neutrophils both locally and systemically, thereby expanding the inflammatory and prothrombotic environment. Thus, NSPs released from activated neutrophils may promote both primary tumors and metastatic tumor expansion.

Extracellular NSPs have been detected in several types of cancer and have been associated with poor clinical outcomes (72). PR3, as well as NE and MPO, were found to be elevated and catalytically active in the urine of patients with bladder cancer, but they were not detected in the urine of healthy controls (177, 178). Due to the presence of neutrophil markers NE and MPO, it is likely that the urinary PR3 originated from cancer-associated neutrophils.

NE has been detected by bronchoalveolar lavage in patients with lung cancer (179). The quantities of NE and MPO in lavage fluid from patients with lung cancer were higher than those in patients with COPD. Studies suggest that NE is involved in primary tumor growth and secondary organ metastasis (180). In mouse models, pharmacological inhibition or genetic deletion of NE reduced cancer progression (78, 180). Consistent with these findings, the cleavage pattern of peptides in cancer tissue was consistent with the cleavage preferences of NE, suggesting that NE is a primary participant in cancer-specific proteolytic processing (181).

In mouse models, sustained lung inflammation induced by lipopolysaccharides or tobacco smoke promoted formation of NETs that awakened dormant malignant mammary cancer cells (73). In this process, activated neutrophils awakened cancer cells by laminin processing via NET-associated NE and MMP-9. The products of proteolytic laminin cleavage triggered transformation of the dormant cancer cells into aggressively growing metastases by activating integrin signaling. In related studies, NETs secreted by inflammation-activated neutrophils impaired tumor clearance by encircling cancer cells and blocking immune cells from exerting their normal anticancer effects (182, 183). However, NE inhibition attenuated these effects, confirming that NE and NETs can mediate the tumorigenic actions of neutrophils and may comprise useful therapeutic targets for multiple types of cancer (73, 180).

Upregulation of DPP1 expression and activity has been observed in human cancer and in animal models of carcinogenesis (184–186). DPP1 expression and enzymatic activity were increased in breast cancer and squamous cell carcinoma in mouse tissue compared with healthy controls (184). In this mouse model, however, DPP1 promoted angiogenesis and tumor growth only in squamous cell carcinoma and not breast cancer. Overall, DPP1 depletion reduced keratinocyte proliferation and vascularization in squamous cell carcinogenesis (184, 187). Interestingly, reduced levels of NE and mast cell chymase were observed in neoplastic skin of DPP1 knockout mice, suggesting a role of immune cell serine protease in squamous cell carcinoma development (184).

An oncogenic role for DPP1 in renal carcinoma (188) and hepatocellular carcinoma has also been reported (186). DPP1 expression was identified as a prognostic marker for survival in humans with hepatocellular carcinoma (186). DPP1 mRNA levels were found to be significantly higher in human hepatocellular carcinoma tissue than in normal healthy tissue, and upregulated DPP1 was correlated with poor overall survival. Interestingly, this study also reported a correlation between high DPP1 expression and clinical features such as cirrhosis and ascites. In gain- and loss-of-function mouse studies, DPP1 was identified as an oncogenic protein that promoted hepatocellular carcinoma cell proliferation and metastasis, potentially involving the TNF-alpha/p38 mitogen-activated protein kinase pathway (186). Collectively, these studies underscore the implications of the wide variation in DPP1 expression and its far-reaching downstream effects, supporting future investigations of DPP1 not only as a biomarker but also as a therapeutic target to combat cancer progression.

Mature DPP1 secreted by tumor cells has shown potential to promote lung metastasis of breast cancer cells (185). DPP1 was identified among the highest regulated proteins, and its expression and secretion were elevated in lung metastases of breast cancer. Moreover, high DPP1 expression in primary tumors was negatively correlated with patients’ overall survival. Hence, tumor-derived DPP1 provides a prognostic survival marker. In addition, in experimental murine breast cancer models, a strong correlation was observed between DPP1 expression and infiltrating neutrophils that deposited NETs in both primary tumors and lung metastasis. The authors reported the dual function of tumor-secreted mature DPP1 in neutrophil recruitment to metastatic niches and NETosis induction.

## 4 Pharmacological inhibition of DPP1

DPP1 is increasingly recognized as a pharmacological target for the blocking of NSPs and NETosis in neutrophil-driven inflammatory and autoimmune diseases (3, 8, 189). Because NE and NETs are identified as therapeutic targets in cancer, pharmacological DPP1 inhibition can also be envisaged as a therapeutic strategy to prevent primary tumor growth and secondary organ metastasis (37). Data from PLS patients-derived neutrophils and monocytes showed abrogated NET formation (162) and strongly reduced intracellular NSP activities and proteins (26, 48, 163). Furthermore, mPR3 on activated neutrophils and monocytes was strongly reduced (48, 163). The observation that DPP1 knockout mice are resistant to NSP-driven experimental diseases supports the therapeutic strategy of DPP1 inhibition (3, 155, 190). The protective effect correlates with the inactivation of NSPs, significantly less secretion of NETs and reduced neutrophil infiltration into the inflammatory sides. Potent reversible as well as irreversible chemical inhibitors blocking DPP1 in the bone marrow have been synthetized, some of which are now being tested in preclinical and clinical trials (3, 13, 189). Pharmacological DPP1 inhibition in neutrophils differentiated from human hematopoietic stem cells recapitulated these findings (48). Consequently, decreased mPR3 levels resulted in reduced PR3-ANCA-induced neutrophil activation. Moreover, pharmacological inhibition reduced neutrophil-induced glomerular microvascular endothelial cell damage (163).

Although small molecule or peptide-based inhibitors against individual NSPs have been extensively explored in the past 50 years, efforts of the pharmaceutical industry so far have created little impact in the clinic, except for AAT augmentation therapy in patients with emphysema and congenital AATD (191). This discovery from the 1960s led to the development of the protease-antiprotease imbalance hypothesis: overshooting protease concentrations, especially high levels of NE, were deemed to have a destructive effect on lung tissue (53). Synergistic involvement of NSPs in tissue damage was demonstrated in mice, with a triple deficiency of NSPs showing better protection against tissue damage than a single NE deficiency in knockout mice (100).

### 4.1 Design and *in vivo* evaluation

Most of the reported nitrile-based inhibitors of DPP1 are dipeptidyl nitriles. The first study, published in 2006 by Bondebjerg et al. (192), started with a moderate DPP1 inhibitor, glycyl-L-phenylalanine nitrile (Gly-Phe-CN). The inhibitory activity towards DPP1 was improved by introduction of aminobutanoic acid (Abu) in the P2 position, whereas various hydrophobic, aromatic amino acid residues, such as phenylalanine (Phe) and biphenyl (Bip), were evaluated in the P1 position. Abu-Bip-CN was identified as the most potent inhibitor in the series (half-maximal inhibitory concentration [IC_50_], 13 ± 3 nM [pIC_50_, 8.7]) but was poorly potent in the cell assay due to rapid proteolytic withdrawal in the cell assay medium. In addition, the amide bond was hydrolyzed in plasma.

The study on Abu-Bip-CN and related compounds was continued and ultimately led to the identification of AZD5248 ((S)-4-amino-N-(1-cyano-2-(4’-cyanobiphenyl-4-yl)ethyl)-tetrahydro-2H-pyran-4-carboxamide), which was selected by Furber et al. as the most promising candidate for *in vivo* studies (36). AZD5248 displayed potent inhibitory activity and selectivity and showed both low clearance and high bioavailability in rat, mouse, and dog models. Although maximal NSP inhibition in bone marrow (90%, 64%, and 88% reduction in NE, PR3, and CatG activity, respectively) was observed in rats after 8 days of treatment (10 mg/kg orally twice daily) (81), AZD5248 showed aortic binding in a rat quantitative whole-body autoradiography study, so its development was stopped (193). The aortic binding was hypothesized to be mediated by imidazolin-4-one formation with aldehydes involved in the cross-linking of elastin, but no direct proof was presented.

A novel series of nitrile inhibitors free from aorta-binding liabilities was developed. Brensocatib (AZD7986/INS1007) was identified as a highly potent, reversible, and selective inhibitor of human DPP1 and showed comparable effects on mouse, rat, dog, and rabbit DPP1. Brensocatib almost completely inhibited activation of NE, PR3, and CatG in a concentration-dependent manner in human primary bone marrow-derived CD34^+^ neutrophil progenitor cells. This compound did not bind to aortic tissue homogenates and showed good stability in plasma with a half-life of greater than 10 h (194). An extensive naïve dosing study with brensocatib at different dosing levels, frequencies, and durations was conducted in rodent models to determine its pharmacokinetic (PK) profile and its pharmacodynamic (PD) effects on NSPs. Dose-dependent PK exposure responses were observed regardless of the rodent species and strain, with mice showing greater NSP activity reduction compared to rats. A duration-dependent reduction was observed in NSP levels that reached a maximum after approximately 7 days and recoveries to baseline levels were nearly symmetrical (195). Brensocatib was shown to mitigate interferon-α-accelerated lupus nephritis in mice (196). Targeting DPP1 with brensocatib was also shown to suppress lung metastasis of breast cancer in mice (185). Furthermore, the combination of brensocatib and anti-PD-L1 antibody was shown to block DPP1-induced colorectal cancer metastasis in mice (197). Brensocatib has also been shown to reduce bone marrow NSP levels, and significantly improve disease score, in two rodent models of rheumatoid arthritis (198).

A preclinical study demonstrated that BI 1291583 can bind human DPP1 in a covalent, reversible manner, selectively and fully inhibiting DPP1 enzymatic activity. This subsequently led to a concentration-dependent inhibition of NE activation in U937 cells and dose-dependent inhibition of NE and PR3 activity (up to 97% and 99%, respectively) in BALF neutrophils in an *in vivo* LPS-challenge model in mice. BI 1291583 distributed to the bone marrow at up to 100 times the exposure compared with plasma (199).

HSK31858 is a potent “non-peptidyl non-covalent” DPP1 inhibitor with an IC_50_ = 57.4 nM. The activities of NSPs all decreased to 15-40% in HSK31858 treated mice versus control mice. This compound exhibited effective anti-inflammatory activity in a rat model COPD (200).

### 4.2 Assessment in clinical trials

To date, several DPP1 inhibitors have progressed to clinical trials in patients with chronic inflammatory lung diseases. In phase 1 studies, brensocatib reduced NE levels in blood with acceptable safety and pharmacokinetics (194, 201), prompting a 24-week phase 2 WILLOW trial conducted in 256 patients with bronchiectasis, in which treatment with 10 mg or 25 mg of brensocatib daily for 24 weeks significantly prolonged time to exacerbations compared with placebo (115, 202). In an exploratory analysis of this study, brensocatib reduced serum NE activity and sputum-associated NSP activity, suggesting a broad anti-inflammatory effect underlying the improved bronchiectasis-associated clinical outcomes observed in the primary trial. Furthermore, positive correlations among the sputum NSPs were observed at baseline and in response to treatment at all time points, pointing to the interrelated efficacy of brensocatib on all three NSP activities (202). The failure of NE inhibitors alone (116), and the data for brensocatib in the Phase 2 WILLOW trial (115, 202), suggests that combined inhibition of NSPs, as well as inhibition prior to extracellular release by the neutrophils, may be required for observation of efficacy in bronchiectasis. Following on from the results of the WILLOW study, a phase 3 study of brensocatib in bronchiectasis is underway (ASPEN; NCT04594369) (203). ASPEN is a multinational, randomized, double-blind, placebo-controlled, parallel-group study that is evaluating the frequency and severity of exacerbations as well as the safety and tolerability of long-term (52-week) treatment with brensocatib compared with placebo in patients with non-cystic fibrosis bronchiectasis. Approximately 1620 participants will be randomized to receive placebo or brensocatib (10 mg or 25 mg) once daily. In addition to this study in patients with bronchiectasis, studies of brensocatib in other indications, such as CRSsNP (NCT06013241), are underway. Another DPP1 inhibitor, BI 1291583, showed generally positive results and an acceptable safety profile in phase 1 studies in healthy volunteers, supporting the initiation of a phase 2 trial in patients with bronchiectasis (Airleaf™; NCT05238675) (204, 205). This multinational, randomized, double-blind, placebo-controlled, parallel-group, dose-finding study has a screening period of at least 6 weeks, a treatment period of 24-48 weeks and a follow-up period of 4 weeks. Approximately 240 adults with bronchiectasis of multiple etiologies will be randomized to placebo once daily, or 3 different doses of BI 1291583 (205). Phase 2 development of HSK31858, a DPP1 inhibitor discovered in China, has also been initiated in patients with bronchiectasis (206).

## 5 Conclusion

NSPs are key mediators of inflammatory lung diseases and other pathologies, and proteolytic damage has been clearly linked to disease progression. Inhibition of DPP1, the key protease that catalyzes maturation of NSPs, is a potential approach to this objective. Consistent with preclinical findings, treatment with a DPP1 inhibitor in a phase 2 study reduced NSP activity and improved outcomes in patients with bronchiectasis. These initial clinical results support the concept of DPP1 inhibition as a potential therapeutic strategy across multiple inflammatory diseases that involve excessive NSP secretion or neutrophil dysregulation and hyperresponsiveness.

## Author contributions

All authors listed have made a substantial, direct, and intellectual contribution to the work and approved it for publication.
